# Mifamurtide and TAM-like macrophages: effect on proliferation, migration and differentiation of osteosarcoma cells

**DOI:** 10.18632/oncotarget.27479

**Published:** 2020-02-18

**Authors:** Francesca Punzo, Giulia Bellini, Chiara Tortora, Daniela Di Pinto, Maura Argenziano, Elvira Pota, Alessandra Di Paola, Martina Di Martino, Francesca Rossi

**Affiliations:** ^1^Department of Woman, Child and General and Specialist Surgery, University of Campania “Luigi Vanvitelli”, Napoli, NA 80138, Italy; ^2^Department of Experimental Medicine, University of Campania “Luigi Vanvitelli”, Napoli, NA 80138, Italy; ^*^These authors contributed equally to this work

**Keywords:** macrophage polarization, tumor micro-environment, osteosarcoma, Mifamurtide, MG63

## Abstract

Tumor-associated macrophages and their alternative activation states together with cytokines and growth factors trapped in tumor microenvironment contribute to the progression of OS. In contrast to other tumor types, M2 polarized macrophages, reduce metastasis and improve survival in osteosarcoma patients. Mifamurtide is an immunomodulatory drug given together with standard adjuvant chemotherapy in high-grade osteosarcoma to improve outcome. Macrophages obtained from peripheral blood mononucleated cells of healthy donors and MG63 cells were cultured alone and together, and treated with Mifamurtide. We analyzed the effects of Mifamurtide on macrophage polarization and on MG63 proliferation, migration and differentiation, evaluating the expression of M1/M2 and osteoblast markers and molecules involved in metastasis and proliferation pathways.

Our data suggest that Mifamurtide, switching macrophage polarization towards a TAM-like intermediate M1/M2 phenotype, may modulate the delicate balance between pro-inflammatory and immunomodulatory macrophage functions. Moreover, Mifamurtide may inhibit the cellular proliferation and induce the tumor cell differentiation, probably through the down regulation of pSTAT3, pAKT and IL-17R.

## INTRODUCTION

Osteosarcoma (OS) is the most common bone tumor of the childhood. Patients who present localized OS have an intermediate prognosis (65% survival rate ADD REF) while patients who presents metastasis have a very poor prognosis [1–3]. The etiology is not completely unraveled however bone niches and their microenvironment seems to have a crucial role in OS development [4]. OS causes increased bone remodeling, resulting from the deregulation of the OPG/RANK/RANK-L pathway [5]. Moreover, in OS, a key osteoblast transcription factor, the Runt Related Transcription Factor 2 (RUNX2), is usually overexpressed [6, 7] and metalloprotease MMP-2, alone or with MMP-9, plays a pivotal role in OS metastases and OS patients’ outcome [8, 9]. Tumor microenvironment (TME) comprises a variety of infiltrating immune cells, including tumor-associated macrophages (TAMs), which are the most abundant [10–13]. TAMs and their alternative activation states greatly contribute to the progression of tumors [14–17].

In response to different stimuli, two distinct forms of activated macrophages can be observed: classically activated macrophages (M1) and alternative activated macrophages (M2). M1 macrophages are activated by Interferon-Gamma (INF-γ) or Lipopolysaccharide (LPS) and exhibit anti-tumor properties through the production of pro-inflammatory cytokines (Interleukin 1 Beta, (IL-1β), Interleukin-6 (IL-6)) and inducible factors against pathogens such as the Tumor Necrosis Factor (TNFα) and the Nitric Oxide Synthase (INOs) [18–22].

M2 macrophages, instead, are activated by anti-inflammatory cytokines (IL-4, IL-10) and the phosphatidiylinositol-3-kinase (PI3K) serine-threonine-kinase (Akt) and mammalian target of Rapamycin (TOR) pathway [23] and exert immunosuppressive effects, enhance angiogenesis and tumor progression. In contrast to many other tumor types, M2 polarized macrophages, reduce metastasis and improve survival in high-grade OS patients. Macrophage polarization is also able to modulate iron sequestration and release [24–28].

M1 macrophages have a high iron content that limits pathogen growth and promotes inflammation. In contrast, M2 macrophages release iron that contributes to cell proliferation [29, 30]. Nevertheless, M1 and M2 macrophages coexist in different activation state, suggesting dynamic changes related to complex tissue-specific signals. Therefore, identify the mechanisms and the molecules involved in macrophage polarization would be useful for both OS diagnosis and therapy. Mifamurtide [liposomal muramyl tripeptide phosphatidylethanolamine (L-MTP-PE)] or MAPACT (Takeda Pharmaceutical Company) is a synthetic analog of a component of bacterial cell walls, which stimulates the immune response activating macrophages and monocytes [31]. Mifamurtide has been approved in Europe for the treatment of non-metastatic OS, in addition to standard chemotherapy [32]. This beneficial combination significantly improves the overall survival of OS patients, reducing the risk of death [33]. According to preliminary clinical report, Mifamurtide is well tolerated and has not severe side effects [34, 35]. Moreover, *in vitro* studies demonstrated that human macrophages can be induced by Mifamurtide to exert anti-tumor activity against OS cells [31]. Based on these evidences, in the present study, we evaluated the role of Mifamurtide in the macrophage polarization and investigated its effects on proliferation, migration and differentiation of OS cells.

## RESULTS

### Mifamurtide directly modulates macrophage polarization

**Figure 1:**
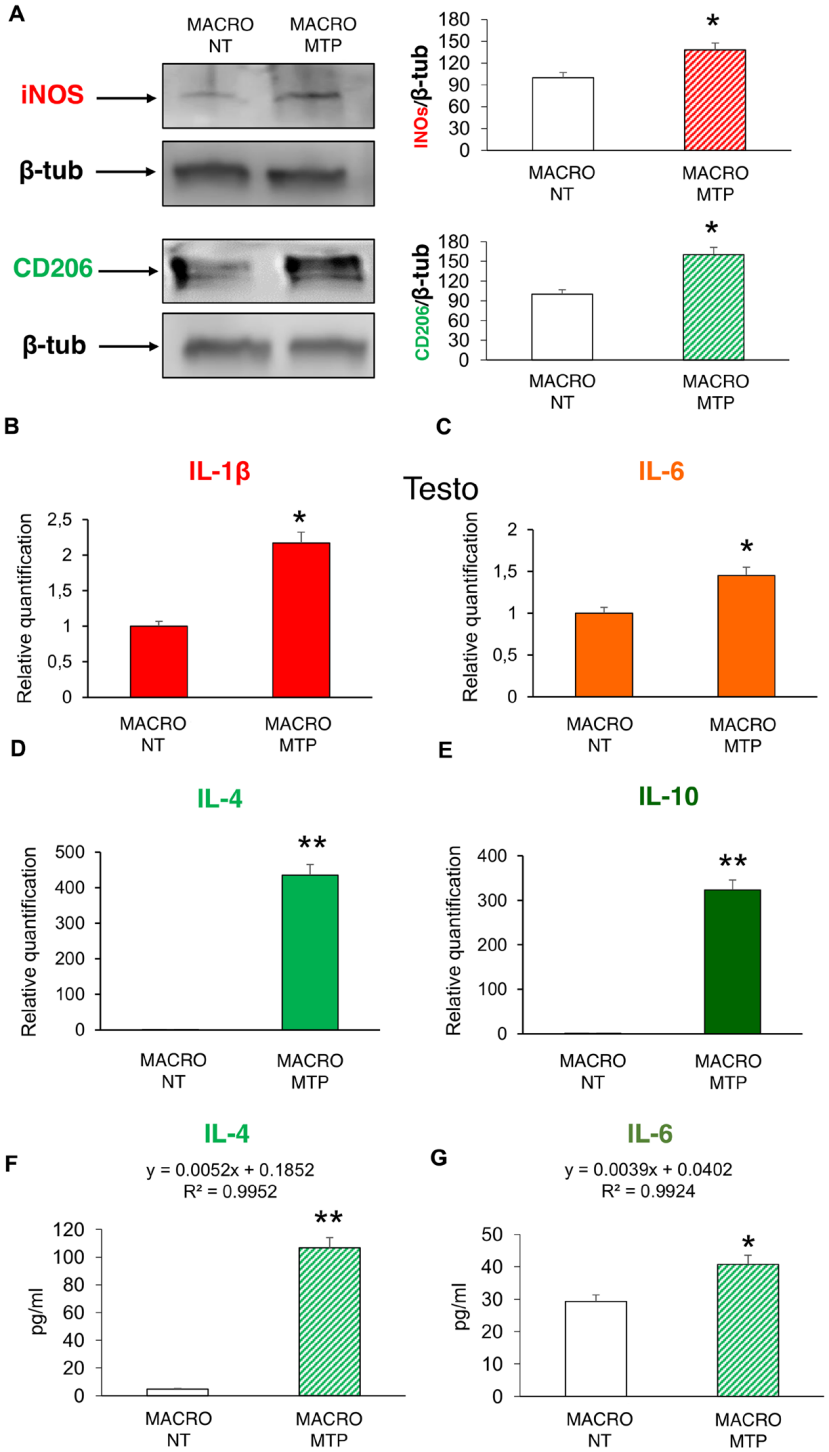
Effects of Mifamurtide (MTP) on macrophage polarization. (**A**) iNOS and CD206 protein expression levels in macrophages activated or not with MTP [100 µM] determined by Western Blot, starting from 15 µg of total lysates. The most representative images are displayed. The proteins were detected using Image Studio Digits software and the intensity ratios of immunoblots compared to untreated (NT) macrophages, taken as 100 (arbitrary unit), were quantified after normalizing with respective loading controls for the housekeeping protein β-Tubulin. The graphs represent the relative quantification for iNOS and CD206 expression as mean ± SD of three independent experiments. A *t*-test has been used to evaluate the statistical differences in protein expression levels. ^*^indicates *p* ≤ 0.05 compared to NT macrophages. (**B–E**) IL-1β, IL-6, IL-4, IL-10 mRNA expression levels in macrophages activated or not with MTP [100 µM] determined by Q-PCR. Results were normalized for the housekeeping gene β-Actin and showed as mean ± SD of three independent experiments. A *t*-test has been used to evaluate statistical differences in mRNA expression levels. ^*^indicates *p* ≤ 0.05 compared to NT macrophages. ^**^indicates *p* ≤ 0.01 compared to NT macrophages. (**F**, **G**) The release of the anti-inflammatory IL-4 and of the pro-inflammatory IL-6 from macrophages activated or not with MTP [100 µM] determined by ELISA assay. The cytokines concentration was determined on a standard concentration curve according to the manufacturer’s instructions. The assays were conducted three times. Data are expressed as mean ± SD (pg/ml). A *t*-test has been used for statistical analysis. ^*^indicates *p* ≤ 0.05 compared to NT macrophages, ^**^indicates *p* ≤ 0.01 compared to NT macrophages.

**Figure 2:**
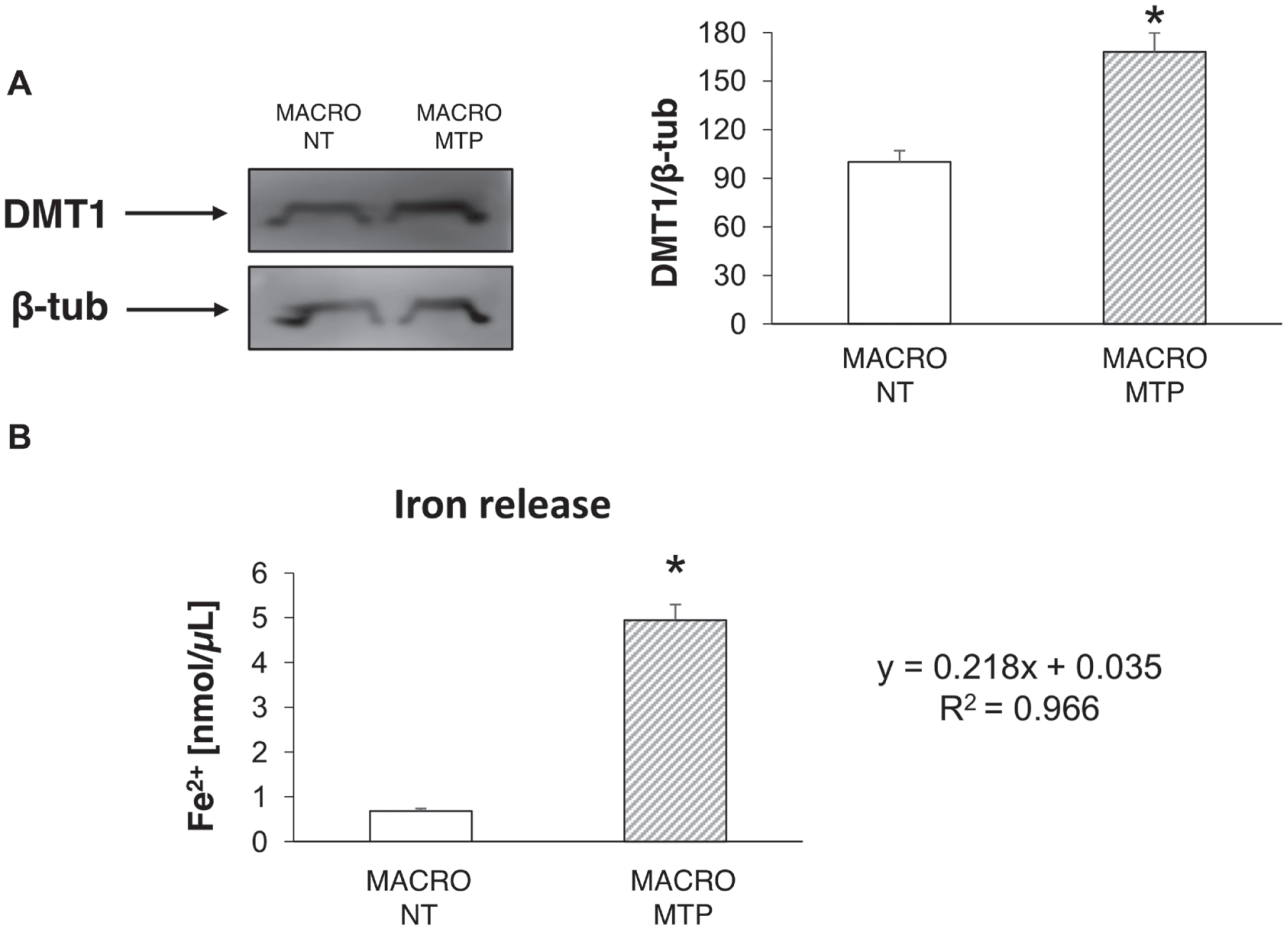
Effects of Mifamurtide (MTP) in macrophages on DMT1 expression and iron release. (**A**) DMT1 protein expression levels in macrophages activated or not with MTP [100 µM] determined by Western Blot, starting from 15 µg of total lysates. The most representative images are displayed. The proteins were detected using Image Studio Digits software and the intensity ratios of immunoblots compared to untreated (NT) macrophages, taken as 100 (arbitrary unit), were quantified after normalizing with respective loading controls for the housekeeping protein β-Tubulin. The graph represents the relative quantification for DMT1 expression as mean ± SD of three independent experiments. A *t*-test has been used to evaluate the statistical differences in protein expression levels. ^*^indicates *p* ≤ 0.05 compared to NT macrophages. (**B**) Fe^2+^ concentration (nmol/μl) measured with the iron assay kit in cell culture supernatants, obtained from macrophages activated or not with MTP [100 µM], determined against a standard concentration curve according to the manufacturer’s instructions. The assay was performed in triplicate. Data are expressed as mean ± SD. A *t*-test has been used for statistical analysis. ^*^indicates *p* ≤ 0.05 compared to NT macrophages.

Mifamurtide-activated macrophages showed a significant increase of both the M1 polarization marker iNOS and the M2 polarization marker CD206 compared to their levels in non-treated macrophages (Figure 1A). Mifamurtide-activated macrophages showed also a significant increase of mRNAs of both pro-inflammatory (IL-1β, IL-6) (Figure 1B and 1C) and anti-inflammatory (IL-4, IL-10) cytokines (Figure 1D and 1E) compared to their levels in non-treated cells. Accordingly, they also showed a massive increase of the release of IL-4 (Figure 1F), and a slight increase of the release of IL-6 (Figure 1G). Moreover, Mifamurtide-activated macrophages showed a significant increase of the iron transporter DMT1 protein (Figure 2A), and a significant increase (more than 7 folds) of the iron release measured with an ELISA assay (Figure 2B).

### Mifamurtide exerts anti-proliferative effect on MG63 cells

**Figure 3:**
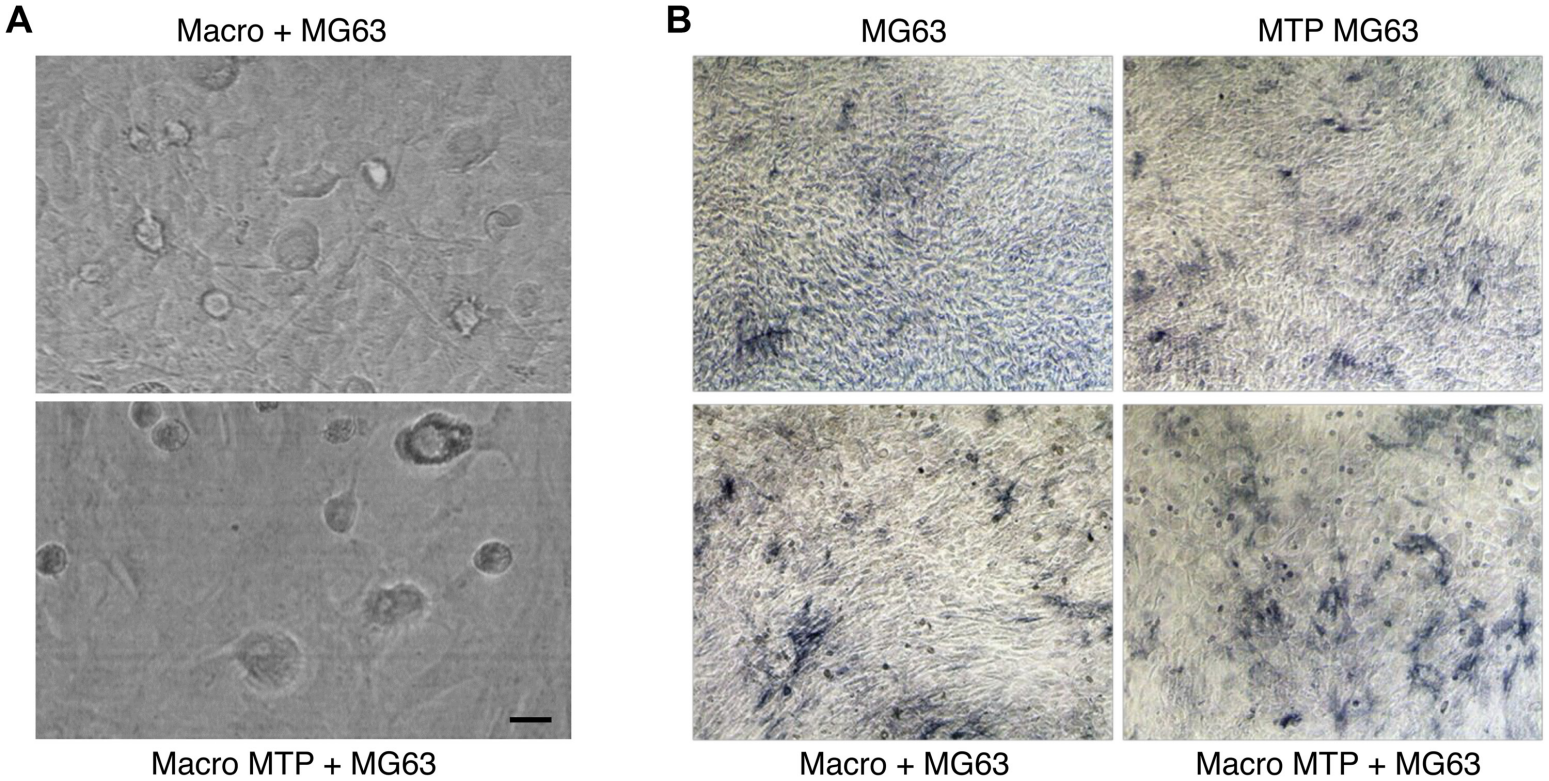
Effects of Mifamurtide (MTP) on MG63 alone and co-cultured with macrophages. (**A**) MG63 in co-culture with macrophages activated or not with MTP [100 µM]. (**B**) ALP colorimetric assay in MG63 alone or in co-culture with macrophages activated or not with MTP [100 µM]. MG63 cells have been seeded in 24 well cell culture plates of fully differentiated macrophages. The representative images, taken on a AE2000 inverted microscope at 10× magnification, are displayed.

Mifamurtide induced a reduction of MG63 cells number when co-cultured with macrophages (Figure 3A). This reduction is even more clear with the colorimetric assay “ALP staining”, which highlighted the direct effect of Mifamurtide on MG63 cells and the macrophage-mediated effects on cells proliferation. This macrophage-mediated effect was in fact more marked when MG63 cells were cultured in presence of Mifamurtide-activated macrophages (Figure 3B).

### Mifamurtide modulates osteoblast markers

**Figure 4:**
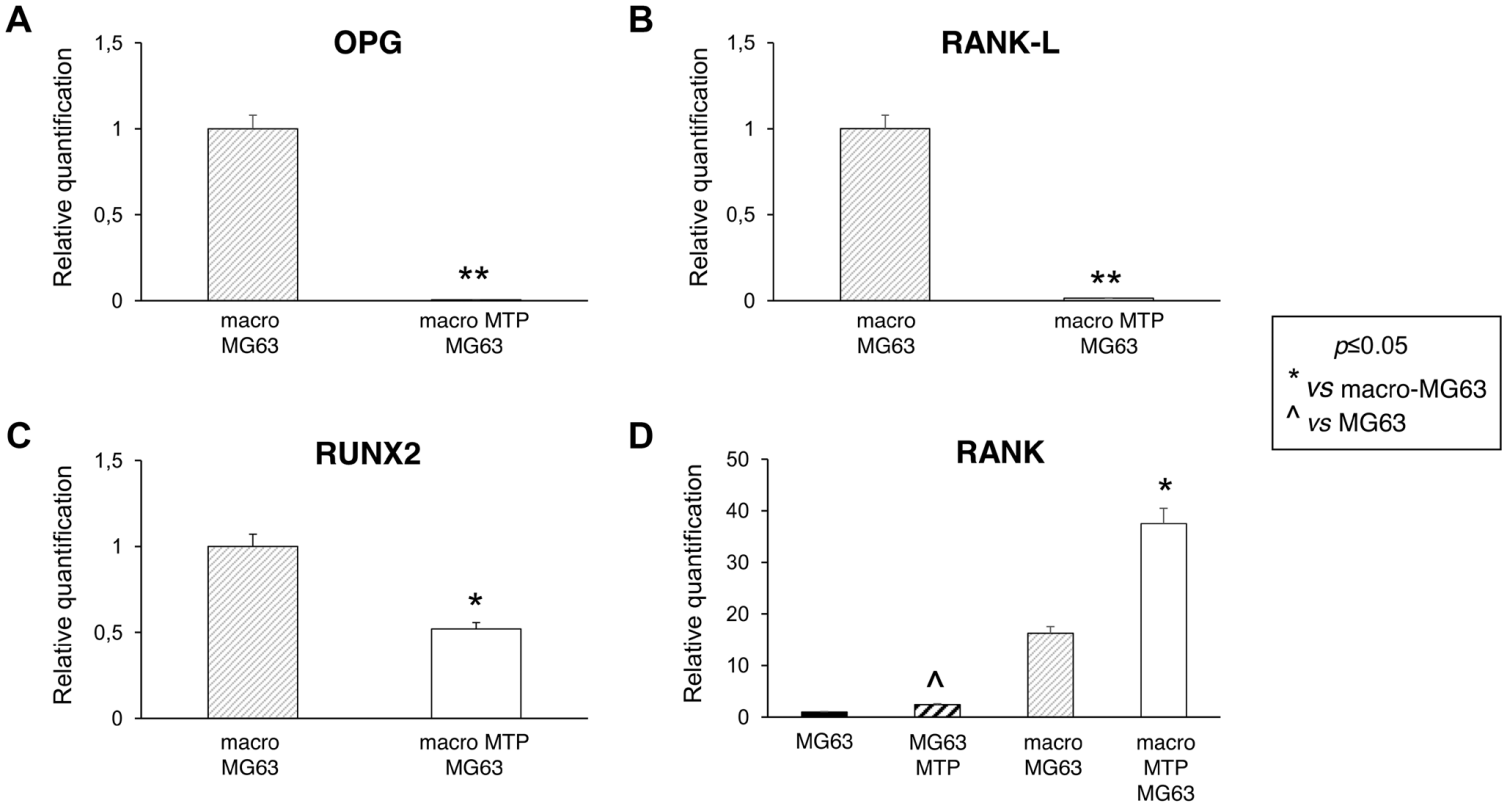
Effects of Mifamurtide (MTP) on bone metabolism markers in MG63 alone and co-cultured with macrophages. (**A**–**C**) OPG, RANK-L, RUNX2 mRNA expression levels in MG63 co-cultured with macrophages activated or not with MTP [100 µM] determined by RT-qPCR. (**D**) RANK mRNA expression levels in MG63 alone or in co-culture with macrophages activated or not with MTP [100 µM] determined by RT-qPCR. Results were normalized for the housekeeping gene β-Actin and showed as mean ± SD of three independent experiments. A *t*-test has been used to evaluate statistical differences in mRNA expression levels. ^*^indicates *p* ≤ 0.05 compared to macrophages-MG63 co-culture, ^**^indicates *p* ≤ 0.01 compared to macrophages-MG63 co-culture, ^ indicates *p* ≤ 0.05 compared to MG63 alone.

MG63 cells co-cultured with Mifamurtide-activated macrophages showed a significant reduction of all the osteoblast markers OPG, RANK-L and RUNX2 (Figure 4A–4C) respect to those co-cultured with non-activated macrophages. Moreover, Mifamurtide induced a significant increase of RANK expression in MG63 cells, both directly and through macrophage activation (Figure 4D).

### Mifamurtide modulates metastatic capacity, prognosis and inflammation markers

**Figure 5:**
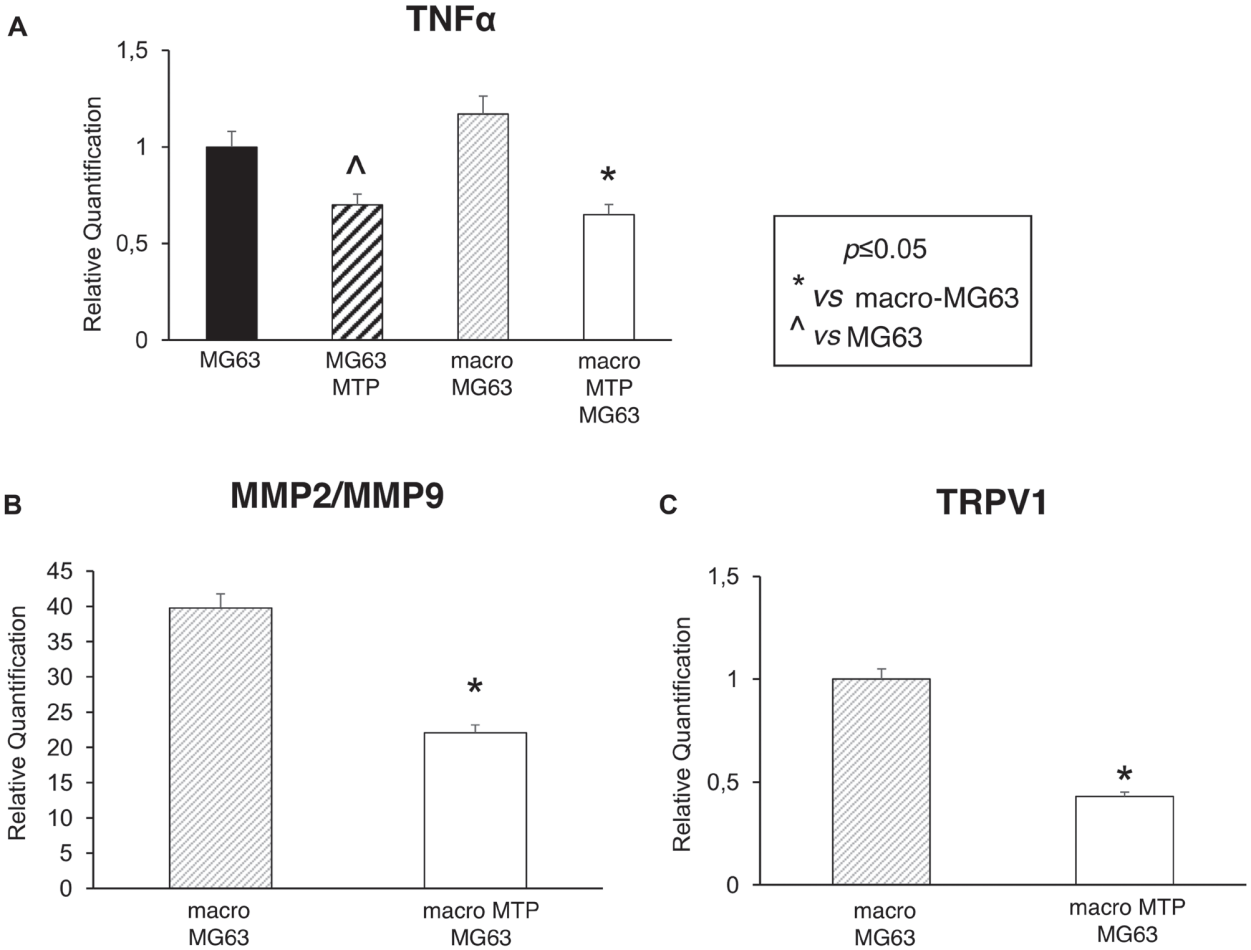
Effects of Mifamurtide (MTP) on TNF-α, MMP2, MMP9 and TRPV1 expression levels in MG63 alone and co-cultured with macrophages. (**A**) TNF-α mRNA expression levels in MG63 alone or in co-culture with macrophages activated with MTP [100 µM] or not determined by RT-qPCR. (**B**, **C**) MMP2/MMP9 and TRPV1 mRNA expression levels in MG63 co-cultured with macrophages activated or not with MTP [100 µM] determined by RT-qPCR. Results were normalized for the housekeeping gene β-Actin and showed as mean ± SD of three independent experiments. A *t*-test has been used to evaluate statistical differences in mRNA expression levels. ^*^indicates *p* ≤ 0.05 compared to macrophages-MG63 co-culture, ^ indicates *p* ≤ 0.05 compared to MG63 alone.

MG63 cells co-cultured with Mifamurtide-activated macrophages show a significant decrease of the metastasis, prognosis and inflammation markers TNFα, MMP2/MMP9, TRPV1 (Figure 5A–5C) compared to those co-cultured with not activated macrophages. The significant reduction of TNFα can be interpreted as the expression of a direct effect of Mifamurtide on MG63 cells (Figure 5A).

### Mifamurtide inhibits pathways involved in osteosarcoma progression

**Figure 6:**
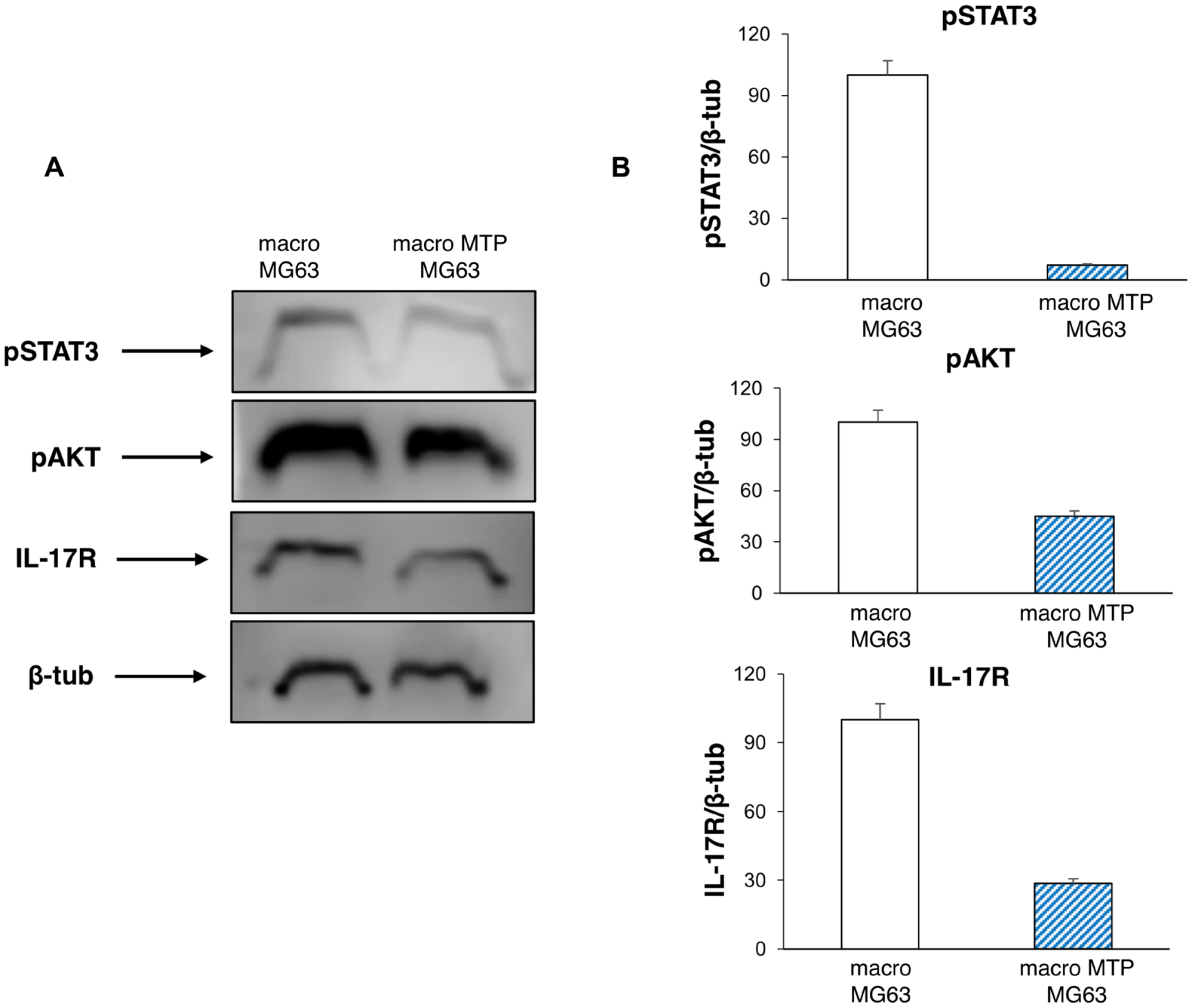
Effects of Mifamurtide (MTP) on proliferation and invasive properties of MG63 co-cultured with macrophages. (**A**) pSTAT3, pAKT, IL-17R protein expression levels in MG63 co-cultured with macrophages activated or not with MTP [100 µM] determined by Western Blot, starting from 15 µg of total lysates. The most representative images are displayed. The proteins were detected using Image Studio Digits software and the intensity ratios of immunoblots compared to macrophages-MG63 co-culture taken as 100 (arbitrary unit), were quantified after normalizing with respective loading controls for the housekeeping protein β-Tubulin. (**B**) The graphs represent the relative quantification for pSTAT3, pAKT, IL-17R expression as mean ± SD of three independent experiments. A *t*-test has been used to evaluate the statistical differences in protein expression levels. ^*^indicates *p* ≤ 0.05 compared to macrophages-MG63 co-culture.

Also the typical markers of cell proliferation are affected by Mifamurtide treatment. In fact, MG63 cells co-cultured with Mifamurtide-activated macrophages show a significant reduction of pAKT and of pSTAT3 with respect to those co-cultured with not activated macrophages (Figure 6A, 6B). Moreover, a significant reduction of the IL-17R protein level, which is an important marker of tumor metastasis, was observed (Figure 6B).

## DISCUSSION

Emerging evidences demonstrate that the tumor microenvironment (TME) plays a crucial role in different steps of OS development: oncogenic transformation, angiogenesis, metastasis, survival and resistance to chemotherapy [11]. TME comprises a variety of infiltrating immune cells, including tumor-associated macrophages (TAMs), which are the most abundant [16]. TAMs and their alternative activation together with cytokines and growth factors are trapped in tumor stroma and strongly contribute to the progression of OS [36]. Interestingly, in contrast to most other tumor types, M2 polarized macrophages reduce metastasis and improve survival in high-grade OS patients [25]. *In vitro* studies demonstrated that human macrophages can be stimulated by Mifamurtide to exert anti-tumor activity against OS cells [31]. In the present study, we evaluated the role of Mifamurtide in macrophages polarization and investigated its effects on proliferation, migration and differentiation of OS cells.

We found that Mifamurtide induces an up-regulation of both surface and release markers of M2 phenotype. Interestingly, we found also an increase of M1 markers, suggesting that Mifamurtide is able to induce a M1/M2 intermediate macrophage phenotype and that macrophage activation state is still ready to promptly cooperate with the immune system. Mifamurtide also modulates the delicate balance of iron, in fact Mifamurtide-activated macrophages show an increase of DMT1, an important iron-uptake transporter [37] and an enhanced iron release. This interesting result suggests that the iron sequestration in the reticuloendothelial system may be actively and efficiently counteracted by the induced iron release, according to an anti-inflammatory profile of the drug.

M1 polarization, resulting in cytotoxicity and inflammation, is induced by STAT1 activation [21]. In contrast, M2 polarization, associated with immune suppression and tumor progression, is induced by STAT3 and STAT6 activation [38]. Interestingly, we found that although inducing M2 macrophage polarization, Mifamurtide exerts a concomitant inhibition of the STAT3 pathway, constitutively activated in OS, reducing STAT3 phosphorylation [39]. The mTOR signaling pathway has been recently implicated in regulating macrophage polarization [40]. The activation of the PI3K/Akt/mTOR pathway promotes anti-inflammatory responses, polarizing macrophages to M2 state [41, 42]. In particular, Akt activation is strongly required. Indeed, Akt inhibition could reduce the M2 genes upregulation [43]. We found that Mifamurtide is able to decrease Akt phosphorylation. Therefore, together with an increase of M2 activation, Mifamurtide seems to limit a long protraction of the M2 activity state.

Cells expressing OPG, RANK and RANK-L are commonly found in tumor microenvironment [44]. OPG/RANK/RANK-L signaling has been shown to play a key role in cancer cell migration and tissue-specific metastatic behavior [45]. In OS this signaling pathway is fully deregulated. In particular, OPG expression is frequently altered in several types of cell tumor including OS cells [46]. Several *in vitro* and *in vivo* studies demonstrated that OPG is able to increase tumor volume and development suggesting that it could serve as survival factor for OS cells and an important marker of cancer progression [47]. Accordingly, we found high levels of OPG and RANK-L in MG63 cells co-cultured with macrophages suggesting the establishment of a vicious cycle between pathological bone remodeling and OS growth.

Noteworthy, MG63 cells co-cultured with Mifamurtide-activated macrophages exhibited a strong reduction of OPG and RANK-L expression according to the anti-osteoporotic effect of the drug previously demonstrated [48]. Moreover, Mifamurtide seems to induce a parallel increase of RANK receptor, probably as a compensatory mechanism restoring the OPG/RANK/RANK-L signaling. Considering that an anti-RANKL therapy could be efficient only in presence of RANK receptor, the increase of the receptor induced by Mifamurtide, is very interesting and confirms Mifamurtide as an important adjuvant for the treatment of OS. This result suggests the possibility of using Mifamurtide in combination with a human monoclonal antibody with high affinity and specificity to RANK-L, for the treatment of OS. Certainly further investigations are needed to investigate the possible synergic interaction between the two compounds. Moreover, Mifamurtide-activated macrophages show a significative reduction of RUNX2 levels breaking the vicious cycle that leads to the exacerbated local bone remodeling. Elevated MMP2/MMP9 ratio is associated with poor response to chemotherapy in OS [49] and Mifamurtide significantly reduces this ratio.

Poor prognosis in oncologic patients is associated with an intense vascularization of primary tumor mass, especially in invasive solid tumors such as OS. Tumor cells secrete angiogenic factors in response to different stimuli, among which, one of the most important is the interleukin 17 (IL-17), a CD4+ T-cell-derived cytokine. A recent study suggested the interleukin 17 receptor (IL-17R) as an important marker of tumor metastasis in OS [50, 51]. Although it has been demonstrated that MG63 compared to the other OS cell lines express the lowest level of IL-17R, we found that Mifamurtide is able to reduce, both directly and through macrophage activation, the expression of IL-17R in MG63 cells.

Collectively, these data suggest that Mifamurtide, switching macrophage polarization towards a TAM-like intermediate M1/M2 phenotype and reducing IL-17R levels and STAT3 activation, may inhibit the cellular proliferation and induce the tumor cell differentiation. Therefore, Mifamurtide might have a double action, on one side, counteracting the pathophysiological switch of TAM toward an M2 immune-suppressive pro-tumoral state into an M1 cytotoxic anti-tumoral activation, and, on the other side, setting the activation state of non-TAM macrophages in a deficient M2 polarization. Thus, an anti-inflammatory but immune-competent setting is maintained with a concomitant down-regulation of Akt phosphorylation, inhibiting the tumor growth and progression.

In conclusion, Mifamurtide seems to interfere with the capacity of macrophages to easily switch from the two opposite activation states M1 and M2, ensuring the maintenance of the delicate balance between pro-inflammatory and immunomodulatory functions of macrophages.

## MATERIALS AND METHODS

### Cell cultures

Macrophages were obtained from peripheral blood mononucleated cells (PBMCs) of 10 healthy subjects. All procedures performed in this study were in accordance with the Helsinki Declaration of Principles and the Ethics Committee of the University of Campania Luigi Vanvitelli, which formally approved the study (Identification code 509, 3 July 2018). Written informed consent was obtained before any procedure. PBMCs were isolated by centrifugation over Histopaque 1077 density gradient (Sigma Chemical, St Louis, MO, USA), diluted in α-Minimal Essential Medium (α-MEM)) (Lonza, Verviers, Belgium) supplemented with 10% fetal bovine serum (FBS) (Euroclone, Siziano, Italy), 100 IU/mL Penicillin, 100 g/mL Streptomycin (Gibco Limited, Uxbridge, United Kingdom), L-Glutamine and seeded in 24-well cell culture plates. In order to obtain fully differentiated human macrophages, the PBMCs were cultured for about 14 days in the presence of 25 ng/mL recombinant human macrophage colony-stimulating factor (rh-MCSF) (Peprotech, London, UK). Macrophages activated (from day 1 until they were fully differentiated) or not with Mifamurtide [100 µM], were simply collected or co-cultured with the most commonly used Human Osteosarcoma cell line, MG63.

MG63 cells were cultured in *Eagle’s* Minimum Essential Medium (EMEM) supplemented with 10% FBS, 100 U/ml Penicillin (Gibco), 100 U/ml Streptomycin (Gibco), 2 mM L-Glutamine (Euroclone) and with 1% Non-Essential Amino Acids (NEAA). At about 70–80% confluence, cells were detached with Trypsin and seeded at the densities of 5,000 cells/well in 24-well cell culture plates of fully differentiated macrophages (at 70–80% confluence) in order to generate co-cultures (21 days). Macrophages and MG63 co-cultures were incubated for 21 days at 37°C in a 5% CO_2_ humidified atmosphere. Monocultures of MG63 were also obtained and treated chronically with Mifamurtide [100 µM] for 7 days.

### RNA extraction, retro-transcription, real time PCR

Total RNA from cultures and co-cultures was extracted using Qiazol^®^ (Qiagen, Hilden, Germany) following the manufacturer’s instructions. EasyScript™ cDNA Synthesis Kit (abm, Foster City, California, USA) was used to synthesize from approximately 1000 ng of mRNA, the first-strand cDNA. The transcript levels of IL-1β, IL-6, IL-4, IL-10, OPG, RANK-L, RUNX2, RANK, TNFα, MMP2, MMP9 and TRPV1 were detected by RT-qPCR using a CFX96 Real-Time PCR system (Bio-Rad, Hercules, CA, USA) using I-Taq Universal SYBR^®^ Green Master Mix (Bio-Rad). The cycling conditions were 10 min at 95°C (initial denaturation) followed by 40 cycles of 15 s at 94°C (denaturation) and 1 min at 68°C (annealing/extension/data collection). The Beta-Actin gene served as the reference gene for the normalization of the real-time PCR products. The PCR primers used to detect each gene were designed using the Primer 3 program and synthesized by Sigma Aldrich. We performed the assays in technical duplicate for each subject and tested the linearity and efficiency of the experiments over dilutions of cDNA including five orders of magnitude. To confirm the specificity of the reactions, we performed the dissociation curve analysis of amplification products. To analyze the data and achieve the relative gene expression levels we used the 2^-DDCt^ method.

### Protein isolation, Western blotting

Protein extraction was performed by using RIPA lysis buffer (Millipore, Italia), according to the manufacturer’s instructions. Total protein concentrations were determined with Bradford dye-binding method (Bio-Rad, Hercules, CA, USA). Total lysates from macrophages, activated or not with Mifamurtide [100 µM] or co-cultured with MG63 cells, were analyzed by Western Blotting. Fifty micrograms of denatured protein were loaded. Membranes were incubated overnight at 4°C with: rabbit polyclonal anti-iNOS (1:1000 dilution; Abcam); rabbit polyclonal anti-CD206 (1:500 dilution; Biorbyt), rabbit polyclonal anti-DMT1 (1:500 dilution; Thermo Fisher Scientific); rabbit polyclonal anti-IL-17R (1:2000 dilution; Abcam); rabbit polyclonal anti-pAKT (1:1000 dilution; Cell Signaling); anti-pSTAT3 (1:1000 dilution; Abcam). Primary antibody incubation was followed by incubation with the relative secondary antibody for 1 h. Reactive bands were detected by chemiluminescence (Immobilon western Millipore) on a C-DiGit^®^ Blot Scanner (LI-COR Biosciences). A mouse polyclonal anti β-Actin antibody (1:1000 dilution; Sigma) was used to check for comparable protein loading as housekeeping protein. Images were captured, stored and analyzed using “Image studio Digits ver. 5.0” software.

### ELISA

#### Cell culture supernatants were obtained from macrophages untreated and treated with Mifamurtide

IL-4 and IL-6 levels were measured using commercially available kits (Human IL-4 ELISA Kit, Thermo Scientific and Human IL-6 Cytokine ELISArray Kit, QIAGEN, respectively), according to the manufacturer’s instructions. Briefly, a microplate was coated with a monoclonal antibody that was specific for IL4 or IL-6. Standards and supernatants were pipetted into the wells of the microplate. A positive control was obtained by pipetting only the standard at different dilutions into the wells. A negative control was obtained by pipetting the standard and cell cultures supernatants into non-coated wells. After the plate was washed, enzyme-linked polyclonal antibodies specific for IL-4 or IL-6 were added to the wells. The reaction was revealed by addition of the substrate solution. The optical density was measured at a wavelength of 450 nm by using the Tecan Infinite M200 (Tecan, Switzerland) spectrophotometer. IL-4 and IL-6 concentrations (pg/ml) were determined against a standard concentration curve.

### Iron assay

Cell culture supernatants, obtained from macrophages activated or not with Mifamurtide, were directly tested to measure released Fe^2+^ iron. The assay was performed by using the Iron Assay Kit (Abcam) according to the manufacturer’s instructions. Briefly, standards and cell cultures supernatants were pipetted into the wells, incubated with an acidic buffer to allow iron release, and then with an iron probe at 25°C for 60 min protected from light. Released iron reacted with the chromogen resulting in a colorimetric (593 nm) product, proportional to the iron amount.

The optical density was measured at a wavelength of 593 nm by using the Tecan Infinite M200 (Tecan, Switzerland) spectrophotometer. The iron release concentration (nmol/µl) was determined against a standard concentration curve.

### Alkaline phospatase (ALP) assay

ALP was evaluated in MG63 cell line by performing a colorimetric assay (Corning Inc., Corning, NY, USA). Cells were harvested in 24-multiwell plates. ALP positivity was quantified using the 5-bromo-4-chloro-3-indolyl phosphate/nitro blue tetrazolium tablet (BCIP/NBT Sigma Fast) that was dissolved in 10 mL of water for the ready-to-use buffered substrate solution. After cell fixation with 10% formalin, the substrate solution, containing BCIP (0.15 mg/ml), NBT (0.30 mg/ml), Tris buffer (100 mM) and MgCl2 (5 mM), pH 9.25–9.75, was added to each well. The removal of the phosphate group by ALP revealed an insoluble indigo product that turned toward intense blue in presence of NBT. The coloration produced was detected with an optical microscope (Nikon Eclipse TS100, Nikon Instruments, Badhoevedorp, The Netherlands). Each experiment included a positive and a negative control to ensure functionality of the assay.

### Drugs

Mifamurtide was diluted in sterile water and used in culture at final concentration of [100 µM] after concentration-response experiments performed on MG63 and macrophages, evaluating cell viability and apoptosis. The drug was tested at five concentrations (1, 10, 100, 200, 500 µM) and the experiments were performed three times. The concentration chosen is the one producing the strongest effect without reducing cells viability (Supplementary Figure 1). Cells viability and apoptosis were evaluated by a fluorometric assay on the Muse^®^ cell analyzer and the results were analyzed with “Muse 1.4 Analysis” software.

### Statistics

All the assays were conducted three times. Data are expressed as mean ± SD. To evaluate differences between quantitative variables a Student’s *t* test was used. Differences were considered statistically significant at *p* ≤ 0.05.

## SUPPLEMENTARY MATERIALS


