# Supplementary material for: Mifamurtide and TAM-like macrophages: effect on proliferation, migration and differentiation of osteosarcoma cells
Source: Oncotarget. 2020 Feb 18;11(7):687–98. doi: 10.18632/oncotarget.27479 (PMC7041936; doi:10.18632/oncotarget.27479)
Supplement: Supplementary file 1 [file oncotarget-11-687-s001.pdf]

Mifamurtide and TAM-like macrophages: effect on proliferation, migration and differentiation of osteosarcoma cells

SUPPLEMENTARY MATERIALS

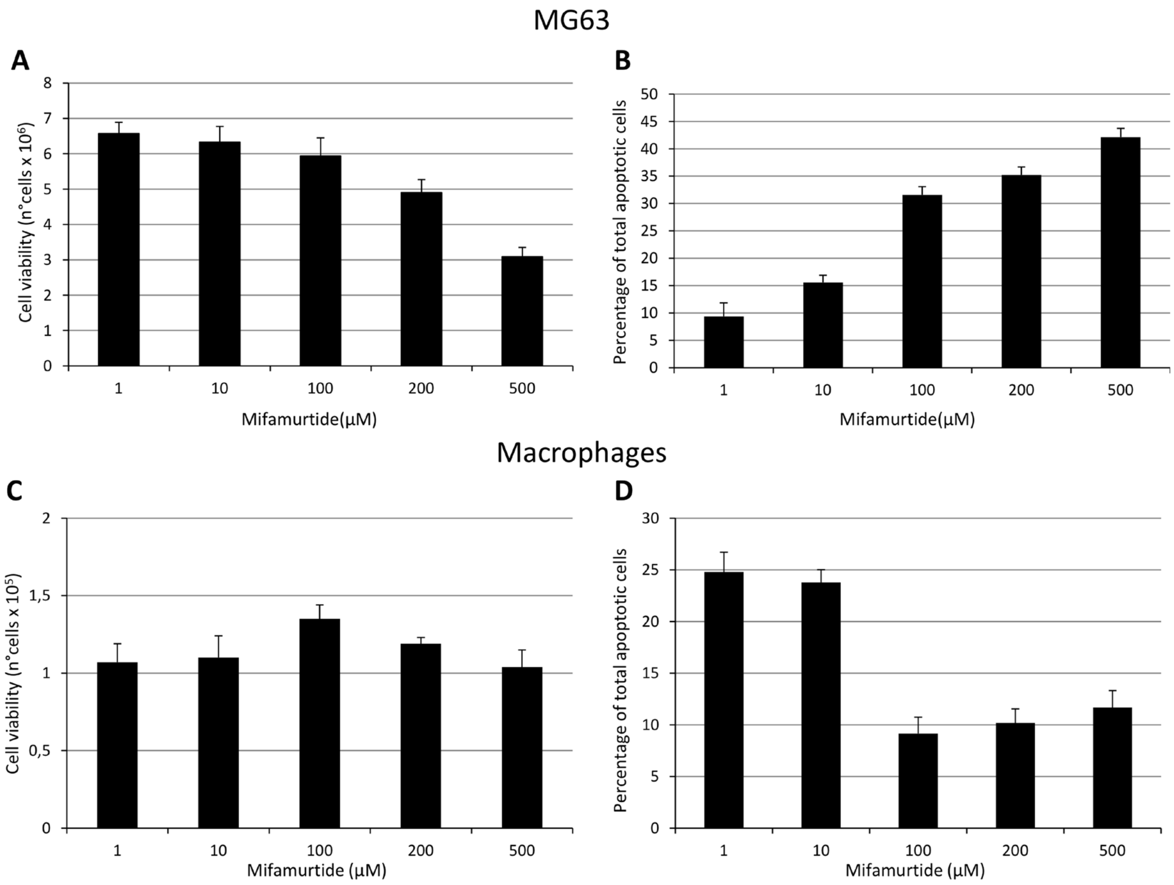

Supplementary Figure 1: Concentration-related effects of Mifamurtide on MG63 cells and on macrophages viability and apoptosis. (**A**, **B**) MG63 cells Viability and Apoptosis (Annexin-V and PI double-stained) Assay was estimated by a cytofluorimetric assay after treatment with Mifamurtide (1, 10, 100, 200, 500 µM). The histograms show results as cell number per 10^6 (MG63) as mean percentage ± SD of three independent experiments. (**C**, **D**) Macrophages Viability and Apoptosis (Annexin-V and PI double-stained) Assay after treatment with Mifamurtide (1, 10, 100, 200, 500 µM). The results are presented as cell number per 10^5 (macrophages) and as the mean percentage ± SD of three independent experiments.
